# Feed Efficiency and Growth Performance in Thai Beef Cattle Fed Cricket Meal as a Soybean Meal Replacement

**DOI:** 10.1155/vmi/6428834

**Published:** 2025-05-21

**Authors:** Benjamad Khonkhaeng, Metha Wanapat, Sarong So, Areerat Lunpha, Ruangyote Pilajun, Pin Chanjula, Pichad Khejornsart, Pongsatorn Gunun, Nirawan Gunun, Bundit Tengjaroenkul, Sineenart Polyorach, Anusorn Cherdthong

**Affiliations:** ^1^Department of Animal Science, Faculty of Agricultural Innovation and Technology, Rajamangala University of Technology Isan, Nakhon Ratchasima Campus, Nakhon Ratchasima 30000, Thailand; ^2^Department of Animal Science, Faculty of Agriculture, Tropical Feed Resource Research and Development Center (TROFREC), Khon Kaen University, Khon Kaen 40002, Thailand; ^3^Department of Animal Science, Faculty of Agriculture and Food Processing, National University of Battambang, Battambang 02352, Cambodia; ^4^Department of Animal Science, Faculty of Agriculture, Animal Feed Research Center, Ubon Ratchathani University, Ubon Ratchathani 34190, Thailand; ^5^Animal Production Innovation and Management Division, Faculty of Natural Resources, Hat Yai Campus, Prince of Songkla University, Songkhla 90110, Thailand; ^6^Department of Agriculture and Resources, Faculty of Natural Resources and Agro-Industry, Kasetsart University, Chalermphrakiat Sakon Nakhon Provinces Campus, Sakon Nakhon 47000, Thailand; ^7^Department of Animal Science, Faculty of Natural Resources, Rajamangala University of Technology Isan, Sakon Nakhon Campus, Sakon Nakhon 47160, Thailand; ^8^Department of Animal Science, Faculty of Technology, Udon Thani Rajabhat University, Udon Thani 41000, Thailand; ^9^Department of Veterinary Public Health, Faculty of Veterinary Medicine, Khon Kaen University, Khon Kaen 40002, Thailand; ^10^Department of Animal Production Technology and Fisheries, Faculty of Agricultural Technology, King Mongkut's Institute of Technology Ladkrabang, Bangkok 10520, Thailand

**Keywords:** ammonia nitrogen, feed conversion ratio, insect, protein replacement

## Abstract

Cricket (*Gryllus bimaculatus*) is a high-protein insect species with a favorable amino acid and fatty acid profile, widely recognized as an alternative to soybean meal in nonruminant diets. However, research on its use in ruminant nutrition remains limited, particularly regarding its effects on feed efficiency and performance. This study evaluated the impact of completely replacing soybean meal with cricket meal on feed intake, nutrient digestibility, rumen fermentation, microbial populations, and growth performance in Thai native beef cattle. Eight male Thai native beef cattle (150 ± 15 kg; ∼2 years old) were used in a completely randomized design with two dietary treatments (*n* = 4 per group). One group received a conventional soybean meal–based diet, while the other received a diet in which 100% of the soybean meal was replaced with cricket meal at an inclusion level of 12% of dry matter. Both diets were formulated to be isonitrogenous and isocaloric. Feed intake was similar between treatments. Crude protein digestibility was higher in the cricket meal group (67.5%) compared to the soybean meal group (63.7%; *p* = 0.04), while other digestibility parameters showed no differences. Blood metabolites, rumen fermentation characteristics, and microbial populations were unaffected by dietary treatment. Cattle fed the cricket meal-based diet showed greater average daily gain (+55.7%; *p* = 0.02) and a 32.9% improvement in feed conversion ratio (*p* = 0.02) compared to cattle fed the soybean meal–based diet. These results suggest that cricket meal can serve as a complete replacement for soybean meal in beef cattle diets, enhancing protein digestibility and growth performance without compromising rumen function.

## 1. Introduction

The rising global demand for animal protein has intensified pressure on livestock feed resources, particularly protein sources [1]. Soybean meal and fish meal, which together account for 60%–70% of total feed costs, pose economic challenges due to price fluctuations and supply constraints [2, 3]. The growing demand has led to a protein shortage, necessitating the exploration of alternative and sustainable protein sources [4, 5]. Additionally, the supply and import prices of conventional feed proteins are subject to fluctuation, further complicating feed management strategies.

Recently, insect-derived protein sources have gained recognition as a promising alternative to traditional protein ingredients like soybean meal and fish meal [3, 6]. Various insects, including black soldier fly larvae, mealworms, and crickets, have been identified as suitable alternatives, offering considerable benefits in animal feed, such as enhanced nutritional value, improved feed efficiency, and positive impacts on reproduction [2, 7]. Among these, cricket (*Gryllus bimaculatus*) is notable for its high crude protein (CP) content (67.7%), ether extract (EE) (13.5%), crude fiber (14.6%), and essential amino acids, including lysine (4.79%), leucine (5.52%), arginine (3.68%), methionine (1.93%), and isoleucine (3.09%) [8–10].

Studies have demonstrated the potential of cricket meal as a replacement for conventional protein sources. Permatahati et al. [11] reported that fish meal could be partially or fully replaced with cricket meal, resulting in increased egg production, egg weight, and yolk quality in layer quails. Boontiam et al. [12] observed that partially replacing fish meal and soybean meal with full-fat cricket meal improved growth performance, intestinal morphology, antioxidant activity, and intestinal microbiota in weaning piglets. Astuti et al. [13] found that replacing 100% of soybean meal with cricket meal in lamb rations did not negatively impact palatability, performance, feed efficiency, or blood metabolites. Similarly, Phesatcha et al. [14] demonstrated that replacing soybean meal with cricket meal pellets (CMP) in concentrate diets improved digestibility, rumen fermentation, and microbial protein synthesis while reducing protozoal populations and methane emissions. However, their study did not assess growth performance in beef cattle, underscoring the need for further research to evaluate these effects and their practical applications for farmers and the livestock industry.

Therefore, this study evaluated the impact of completely replacing soybean meal with cricket meal on feed intake, nutrient digestibility, rumen fermentation, microbial populations, and growth performance in Thai native beef cattle.

## 2. Materials and Methods

### 2.1. Cricket Meal Preparation

Fifty-day-old crickets were collected, washed, and processed in a hot-air oven at 100°C for 30 min, then dried at 65°C for 48 h to remove excess moisture. The dried crickets were ground to a 1-mm particle size for further nutrient analysis.

The nutrient compositions of the cricket meal were analyzed for dry matter (DM), organic matter (OM), nitrogen, EE, and ash, following AOAC methods [15]. The nitrogen content was used to calculate CP by multiplying it by a factor of 6.25. The fiber content, including neutral detergent fiber (NDF) and acid detergent fiber (ADF), was determined using an Ankom200 Fiber Analyzer (Ankom Technology, Macedon, NY, USA) following the procedure described by Van Soest et al. [16]. This system allows for an automated, closed-bag digestion process, ensuring consistency and accuracy in fiber determination. The nutrient compositions of the cricket meal are summarized in Table 1. The diets were formulated to be isonitrogenous, containing 15.4%–15.8% CP on a DM basis, and isocaloric, with metabolizable energy (ME) levels ranging from 13.3 to 13.8 MJ/kg DM, in accordance with recommended nutrient requirements. Cricket meal, derived from *G. bimaculatus*, was used as a replacement for soybean meal in this study. The chemical composition of *G. bimaculatus* includes 52.1% CP, 27.7% EE, 23.3% NDF, 12.7% ADF, and an estimated ME value of 20.1 MJ/kg DM.

### 2.2. Animals, Experimental Design, and Experimental Procedures

The Institutional Animal Care and Use Committee of Khon Kaen University (KKU) confirmed all of the procedures using animals in the metabolism experiments (Record IACUC-KKU-93/64). Eight male Thai native beef cattle, approximately 2 ± 0.3 years of age, with an average body weight of 150 ± 15 kg, were used in this study. Prior to the experiment, all animals were treated with ivermectin combined with 10% Clorsulon (Merial Animal Health Co. Ltd., Nanjing, China) to eliminate internal and external parasites. The cattle were housed individually in pens measuring 2 × 3 m, with unrestricted access to clean drinking water and mineral blocks. Animals were assigned to a completely randomized design with two dietary treatments (*n* = 4 per group): One group received a control diet containing soybean meal, while the other received an experimental diet in which 100% of the soybean meal was replaced with cricket meal at an inclusion level of 12% of DM. This substitution level was selected to evaluate the full replacement potential of cricket meal as a protein source in place of soybean meal, while maintaining dietary protein equivalency. We selected the 100% substitution level based on prior in vitro findings, suggesting the feasibility of full replacement without adverse effects on digestibility and fermentation [14]. Testing at the maximum inclusion level provides a benchmark for evaluating performance limits and practical application in resource-limited systems. However, we acknowledge that intermediate levels (e.g., 33%, 66%) could help identify optimal inclusion rates and recommend such dose–response evaluations for future studies. The concentrate diets were formulated to be isonitrogenous, with cricket meal and soybean meal both included at 12% of DM, ensuring that the overall CP content met the nutritional requirements for Thai native beef cattle, as shown in Table 1. Concentrate was offered at 1% of body weight (DM basis), while rice straw was provided ad libitum to ensure a minimum of 10% refusal. Diets were fed twice daily at 07:00 and 16:00. Body weights were recorded every two weeks to adjust feed offered throughout the 90-day experimental period. The chemical composition of both concentrate and rice straw is provided in Table 1.

Although the sample size was limited to eight animals due to facility constraints, care was taken to ensure homogeneity in sex, age, and body weight across groups. Despite this limitation, statistically significant differences were observed in key outcomes, including CP digestibility, average daily gain (ADG), and feed conversion ratio (FCR). To aid interpretation, effect sizes and 95% confidence intervals were reported for major performance variables to demonstrate the magnitude and precision of treatment effects. Similar sample sizes have also been effectively used in top-tier studies on ruminant growth and digestibility [17–19].

### 2.3. Sampling and Analysis

Cattle were allowed a 14-day adaptation period to the dietary treatments before sampling commenced. Feed offered and residues were recorded daily throughout the 90-day experimental period to calculate daily feed intake and FCR. On the final day of the feeding period, cattle were weighed to determine final body weight, which was then used to calculate the ADG.

Weekly samples of concentrate and rice straw were collected, pooled, and stored at −40°C until analysis. Prior to analysis, the samples were thawed, dried in a hot-air oven at 100°C for 24 h, and ground to a 1-mm particle size for chemical composition analysis. The samples were analyzed for DM, OM, nitrogen, EE, and ash using AOAC methods [15]. CP was calculated by multiplying the nitrogen content by 6.25. NDF and acid ADF were analyzed using an Ankom analyzer following the method of Van Soest et al. [16].

In the final 7 days of the experiment, cattle were transferred to metabolism crates to assess apparent nutrient digestibility. Feces were collected using a total collection method. Feces were pooled by individual cattle, frozen at −40°C, and later thawed, oven-dried at 100°C for 48 h, and ground to a 1-mm particle size for analysis. The fecal samples were analyzed for DM, OM, nitrogen, EE, and ash using AOAC methods [15].

After the collection of feces, cattle were returned to their pens for blood and ruminal fluid sampling. Blood samples (5 mL) were drawn from the jugular vein at 0 h before and 4 h after the morning feeding into test tubes containing EDTA to prevent coagulation. Plasma was separated by centrifugation at 500× *g* for 10 min and stored at −20°C for analysis. Blood urea nitrogen (BUN) and blood glucose concentration were determined using a diagnostic kit (Albumin-HRII, L type Wako UN, Glucose-HRII, Wako Diagnostics, Mountain View, CA, USA).

Ruminal fluid samples were collected via a vacuum pump at 0 h before and 4 h after morning feeding. The pH and temperature of the ruminal fluid were immediately measured using a Hanna pH meter (Hana Instruments, Nusfalău, Romania). Ruminal fluid was divided, and 45 mL was mixed with 5 mL of 1 mol/L sulfuric acid to prevent nitrogen loss and stored at −20°C for analysis. This portion was used to determine ammonia nitrogen (NH_3_-N) using the method of AOAC [15] and to analyze volatile fatty acids (VFAs), including acetic acid (C2), propionic acid (C3), and butyric acid (C4), following the procedure of Yamamoto-Osaki et al. [20] using gas chromatography (model HP6890, Hewlett-Packard, CA, USA, column Restek 1207384, Stabilwax 60°C–250°C, 30 m × 250 μm × 0.25 μm). The second portion (1 mL) was mixed with 9 mL of 3 mol/L formalin to study microbial populations, including bacteria, protozoa, and fungal zoospores. Microbial counts were determined using a hemocytometer slide (Boeco, Hamburg, Germany) following the method modify by So et al. [17].

### 2.4. Statistical Analysis

All data were analyzed using a completely randomized design in SAS Statistics version 9 [21]. The statistical model used was as follows:

(1) $$\begin{array}{c} Y_{ij}=\mu +\tau_{i}+\varepsilon_{ij}, \end{array}$$

where *Y*_*ij*_ represents the observed value, *μ* is the overall mean, *τ*_*i*_ is the effect of treatments, and *ε*_*ij*_ is the residual error. Prior to analysis, data distribution was checked using the Proc Univariate function in SAS. The data were reported as an average from four steers with the standard error of means. Statistical significance was considered at *p* < 0.05.

## 3. Results

### 3.1. Feed Intake and Digestibility

Feed intake and nutrient digestibility of beef cattle fed soybean meal and cricket meal as protein sources are presented in Table 2. There were no differences in feed intake between the two dietary treatments. Cattle offered either the soybean meal–based or cricket meal–based diet consumed comparable amounts of rice straw (2.8 kg DM), concentrate (1.4 vs. 1.5 kg DM), and total DM (4.2 vs. 4.4 kg DM), representing 2.8% and 2.9% of BW, respectively. In terms of nutrient digestibility, CP digestibility was higher (*p* = 0.04) in cattle fed the cricket meal–based diet (67.5%) compared to those fed the soybean meal–based diet (63.7%). However, the digestibility of DM, OM, NDF, and ADF showed no differences between treatments.

### 3.2. Blood Metabolites and Ruminal Fermentation Characteristics

Blood metabolites and rumen fermentation characteristics of beef cattle fed soybean meal–based and cricket meal–based diets are presented in Table 3. There were no differences in any of the blood metabolite or rumen fermentation parameters between the two dietary groups. BUN (12.1 vs. 13.5 mg/dL), blood glucose (75.3 vs. 74.6 mg/dL), and ruminal NH_3_-N (16.2 vs. 18.4 mg/dL) were comparable between cattle fed the soybean meal and cricket meal diets. Similarly, ruminal pH (6.9 in both groups) and temperature (39.2°C vs. 39.0°C) were unaffected by diet.

Although total TVFA concentrations were numerically higher in the cricket meal group (99.1 vs. 97.7 mmol/L), the difference was not statistically significant. The molar proportions of individual VFAs—including C2, C3, and C4—also showed no variation between the two treatments.

Microbial populations, expressed as log_10_ cells/mL, were similar across diets. Bacterial counts (10.5), protozoal counts (6.9), and fungal zoospore counts (4.4 vs. 4.5) did not differ, suggesting that full replacement of soybean meal with cricket meal had no adverse effects on the rumen microbial ecosystem.

### 3.3. Cattle Performance

Growth performance of beef cattle fed soybean meal– and cricket meal–based diets is presented in Table 4. Initial body weight did not differ between treatments. However, cattle receiving the cricket meal–based diet showed notable improvements in performance indicators. Final body weight increased by 10.7% compared to the soybean meal group (*p* = 0.04), while body weight gain improved by 55.6% (*p* = 0.03).

Moreover, ADG was 55.7% higher in the cricket meal group (721.1 ± 20.5 g/day vs. 463.3 ± 20.5 g/day, *p* = 0.02; Cohen's *d* = 3.2, 95% CI: 1.1–5.1), indicating enhanced growth rate. FCR also improved by 32.9% (6.1 vs. 9.1, *p* = 0.02; Cohen's *d* = −2.7, 95% CI: −4.6 to −0.8), reflecting greater feed efficiency.

## 4. Discussion

As shown in Table 1, *Gryllus bimaculatus* (cricket) contained 52% CP (DM basis) and 27.7% fat. Udomsil et al. [22] reported that *Gryllus bimaculatus* contained 60% CP (DM basis), which is 8% higher than the value observed in this study, and a lipid content of 23.4%. Similarly, Phesatcha et al. [14] noted that *Gryllus bimaculatus* contained 16.5% more CP than reported in this study and 8.5% more than the value reported by Udomsil et al. [22], as well as 12.5% more fat. Variations in the CP content of *Gryllus bimaculatus* are likely influenced by factors such as age, feed quality, and rearing environment [23–25].

Insects have been increasingly recognized as a promising alternative protein source to soybean meal and fish meal [3]. In monogastric animals, diets where cricket meal partially or completely replaced soybean meal had improved growth performance and feed efficiency [8, 12]. Nonetheless, studies evaluating cricket meal as a replacement for soybean meal in ruminant diets, particularly regarding growth performance, are still scarce.

Replacing 100% of soybean meal with cricket meal (12% DM) did not affect feed intake, indicating no adverse effects on palatability. Similar results were observed in goats, where cricket meal inclusion had no significant impact on intake in both preweaning and postweaning diets [26]. In lambs, Astuti et al. [13] observed that replacing soybean meal with cricket meal up to 100% (15% DM) in rations also did not affect feed intake.

In the present study, nutrient digestibility parameters, including DM, OM, and fiber fractions, were not affected by the replacement of soybean meal with cricket meal. However, CP digestibility was higher in cattle fed the cricket meal diet. This improvement is likely due to the favorable protein quality and amino acid profile of *Gryllus bimaculatus*, which may support more efficient ruminal digestion and postruminal absorption [14]. Additionally, insect protein is known for its high solubility and digestibility at moderate inclusion levels [27]. Supporting this, Phesatcha et al. [14] reported that replacing soybean meal with CMP improved the digestibility of CP, NDF, and ADF, while DM and OM digestibility remained unchanged. In contrast, other studies have reported reduced digestibility when insect meals were used to replace conventional protein sources. For example, Jayanegara et al. [10, 28] and Astuti et al. [13] observed lower DM and OM digestibility in ruminant diets containing cricket meal, likely due to the presence of chitin. However, differences in insect species, inclusion levels, and experimental design may account for these inconsistencies. Taken together, our findings suggest that cricket meal at 12% DM can improve CP digestibility without compromising other aspects of nutrient digestion, supporting its potential as an effective alternative protein source in beef cattle diets.

Furthermore, Hervás et al. [4] explored the use of house cricket (*Acheta domesticus*) meal (HCM) as a replacement for soybean meal in ruminant diets. Comparing a control diet with 10% SBM to a test diet with 10% nondefatted HCM, they found that HCM inclusion enhanced ruminal lipid metabolism. This led to higher concentrations of beneficial fatty acids, such as trans-11 18:1 and cis-9 trans-11 conjugated linoleic acid (CLA), while maintaining stable biohydrogenation pathways with minimal changes in the trans-10:trans-11 18:1 ratio.

Complete replacement of soybean meal with cricket meal did not significantly alter BUN concentrations, with values recorded at 12.1 mg/dL and 13.5 mg/dL for the soybean and cricket meal groups, respectively. Both values remained within the optimal physiological range (> 7 mg%) for supporting effective microbial protein synthesis in the rumen [27, 29]. Although BUN was numerically higher in the cricket meal group, this difference was not statistically significant and may reflect normal biological variation. Similarly, NH_3_-N concentrations showed no significant treatment effects. The lack of significant differences in BUN and ruminal NH_3_-N concentrations between treatments is consistent with the isonitrogenous formulation of the diets. When the CP content is balanced and nitrogen supply is synchronized with available fermentable energy, ammonia utilization by rumen microbes is generally stable, resulting in comparable nitrogen metabolites across treatments [4, 17]. While previous in vitro studies have reported increased NH_3_-N levels in cricket meal–based substrates—such as those by Ahmed et al. [30] and Phesatcha et al. [14]—our findings suggest that, under the present in vivo conditions, ruminal NH_3_-N levels remained comparable between diets. Therefore, any interpretation regarding enhanced nitrogen release or utilization from cricket meal should be approached with caution and further validated in studies with greater statistical power.

Replacing soybean meal with cricket meal at 100% DM reduced blood glucose levels from 75.3 mg/dL to 74.6 mg/dL, which is still above the optimal range of 40–60 mg/dL, indicating a positive energy balance [31, 32]. Limited studies are available on blood glucose levels in ruminants fed diets containing cricket meal. However, Astuti et al. [13] observed a decrease in blood glucose from 66.22 mg/dL to 58.04 mg/dL when soybean meal was replaced with cricket meal at 100% DM in lamb diets, although the difference was not statistically significant.

Ruminal pH (6.9) and temperature (39°C) in both diet groups were within the optimal ranges. Similarly, Phesatcha et al. [14] reported that substituting cricket meal for soybean meal at 100% DM did not affect in vitro ruminal pH, although their reported values were 0.2–0.5 units lower than those in the current study. Although the molar proportions of VFA (C2, C3, and C4) were not different between treatments, a numerical increase in total VFA concentration was observed in the cricket meal group (99.1 mmol/L) compared to the soybean meal group (97.7 mmol/L). While differences were not statistically significant, the numerically higher total VFA concentration in the cricket meal group suggests a potential metabolic response worth further investigation. The higher EE content (6.5% DM vs. 3.4% DM) may have influenced microbial activity, as elevated dietary fat has been reported to affect rumen microbial populations and fermentation patterns [33–35]. Enjalbert et al. [34] demonstrated that specific fatty acids, such as palmitic and stearic acid, can inhibit microbial species like *Prevotella ruminicola* and *Butyrivibrio fibrisolvens*, which are associated with VFA production. While specific microbial profiles were not assessed, the presence of palmitic (9.3%) and stearic acid (2.7%) in cricket meal—higher than in soybean meal—may have influenced fermentation dynamics. Nonetheless, due to the lack of significant changes in VFA profiles, these speculations require validation through microbial sequencing and targeted fermentation assays [23, 36]. Similar numerical reductions in individual VFA proportions with cricket meal inclusion have been reported by Astuti et al. [13] in lambs and by Phesatcha et al. [14] in Thai native cattle. However, interpretations remain limited due to the lack of significant changes and the influence of additional dietary factors such as roughage-to-concentrate ratios. For example, Astuti et al. [26] reported a nonsignificant increase in acetate concentration with increasing cricket meal levels, potentially due to chitin-derived fiber. Overall, while the current findings do not show statistical differences, they underscore the need for future studies using larger sample sizes and microbial profiling techniques to explore the effects of insect-derived lipids on rumen fermentation dynamics.

The bacterial population in the current study was 10.53 log_10_ cells/mL, which falls within the optimal range of 10.0–11.0 log_10_ cells/mL as reported by Matthews et al. [37]. In postweaning goats, Astuti et al. [26] observed lower bacterial populations, ranging from 9.98 to 9.99 log_10_ cells/mL, likely due to differences in animal age, rumen development, and dietary composition. Protozoal populations in both treatment groups were also within the normal physiological range (> 6.0 log_10_ cells/mL) as reported by Michaiowski [38]. Phesatcha et al. [14] found that increasing levels of cricket meal substitution for soybean meal decreased protozoal counts in vitro, from 5.96 to 5.79 log_10_ cells/mL. This effect was influenced by both cricket meal inclusion and the roughage-to-concentrate ratio, indicating a complex interaction affecting protozoal populations. Similarly, Astuti et al. [26] reported a reduction in protozoal populations from 6.25 to 5.74 log_10_ cells/mL with increasing levels of cricket meal in diets for postweaning goats.

Previously, research on cricket meal supplementation or its replacement for soybean meal in ruminant diets has been limited, particularly with respect to growth performance. Phesatcha et al. [14] reported that replacing soybean meal with CMP form improved digestibility, rumen fermentation, and microbial protein synthesis while reducing methane emissions. However, the scarcity of data on growth performance, particularly in cattle, emphasizes the need for further investigation—especially considering the physical form of the cricket meal used.

The present study addresses this gap by evaluating the impact of completely replacing soybean meal with cricket meal at 12% DM on feed efficiency and growth performance in Thai native beef cattle. Results showed that cattle fed the cricket meal-based diet had a final body weight of 210.5 kg, compared to 190.1 kg in the soybean meal group—an increase of 10.7%. Correspondingly, body weight gain increased from 41.7 to 64.9 kg (a 55.6% improvement) and ADG increased from 463.3 to 721.1 g/day (+ 55.7%). Additionally, FCR improved from 9.1 to 6.1, representing a 32.9% increase in feed efficiency. The observed improvement in growth performance in cattle fed the cricket meal–based diet is most plausibly linked to the significantly enhanced CP digestibility. Enhanced protein utilization likely provided greater amino acid availability to support tissue accretion. While previous studies have reported microbial shifts and nitrogen metabolism changes with insect-based feeds [14, 30], no significant differences in NH_3_-N or BUN levels were observed in this study. Therefore, in the absence of direct microbial or enzymatic assessments, such interpretations remain speculative and should be approached with caution. Further research is warranted to elucidate the precise mechanisms behind these performance improvements.

Cricket meal may also offer favorable protein fractions with higher solubility and digestibility. This aligns with findings by Narang and Lal [39], who reported increased CP digestibility and body weight gain in Jersey calves when silkworm pupae replaced groundnut cake as a protein source. Additionally, the protein quality and postruminal digestibility of cricket meal likely contribute to its performance benefits. Toral et al. [40] demonstrated that *Acheta domesticus* exhibited high nitrogen solubility and comparable intestinal digestibility of undegraded nitrogen to soybean meal, supporting efficient amino acid absorption beyond the rumen.

In contrast, Astuti et al. [13] found that replacing 15% DM of soybean meal with cricket meal in lamb diets reduced apparent protein digestibility, ADG, and feed efficiency. These discrepancies may result from differences in animal species, physiological stage, and diet composition, highlighting the need for further research to confirm and extend the present findings in beef cattle.

## 5. Conclusion

This study provides evidence that cricket meal (*Gryllus bimaculatus*) can serve as a viable alternative to soybean meal in beef cattle diets, with potential nutritional and performance advantages. When included at 12% of DM to fully replace soybean meal in the diet, cricket meal enhanced CP digestibility, ADG, final body weight, and FCR. These effects were supported by clear percentage improvements in performance indicators, underscoring practical relevance even in a small cohort. These improvements in growth performance suggest that cricket meal could be a practical and sustainable protein source for cattle producers, particularly in regions where conventional protein feeds are expensive or limited. To build on these encouraging results, future studies involving larger animal populations and extended feeding durations are essential. Such research will help confirm the consistency of these findings, explore variability among different production settings, and evaluate the long-term effects of cricket meal on cattle productivity, health, and environmental sustainability.

## Figures and Tables

**Table 1:** Feed ingredients and chemical composition of concentrates and cricket meal (*Gryllus bimaculatus*).

Item	Soybean meal–based diets	Cricket meal–based diet	Cricket meal composition
Cassava chip	50.0	50.0	—
Rice bran	15.0	15.0	—
Soybean meal	12.0	—	—
*Gryllus bimaculatus*	—	12.0	—
Palm kernel meal	17	17	—
Molasses	2.3	2.5	—
Sulfur	1.0	1.0	—
Urea	1.5	1.0	—
Premix^†^	1.0	1.0	—
Salt	1.0	1.0	—
Chemical composition			
Dry mater (%)	84.9	85.0	91.0
Organic matter (%DM)	92.3	93.2	93.0
Crude protein (%DM)	15.4	15.8	52.1
Ether extract (%DM)	3.4	6.5	27.7
NDF (%DM)	38.6	37.2	23.3
ADF (%DM)	17.1	17.2	12.7
ME (MJ/kg DM^∗^)	13.3	13.8	20.1

Abbreviations: ADF = acid detergent fiber, DM = dry matter, and NDF = neutral detergent fiber.

^†^Premix (per 1 kg) composed of vitamin A 4 × 10^6^ IU, vitamin D3 4 × 10^5^ IU, vitamin E 4000 IU, vitamin B12 0.002 g, Mn 16 g, Fe 24 g, Zn 10 g, Cu 2 g, Se 0.05 g, Co 0.2 g, and I 0.5 g.

^∗^Calculated value.

**Table 2:** Feed intake and digestibility of beef cattle fed soybean meal and cricket meal as protein sources.

Item	Soybean meal–based diets	Cricket meal–based diet	SEM	*p* value
Feed intake				
Rice straw (kg DM)	2.8	2.8	0.13	0.87
Concentrate (kg DM)	1.4	1.5	0.09	0.54
Total intake (kg DM)	4.2	4.4	0.23	0.71
% BW	2.8	2.9	0.12	0.51
Nutrient digestibility (%)				
Dry matter	69.8	68.4	1.44	0.98
Organic matter	70.2	69.1	1.89	0.47
Crude protein	63.7	67.5	0.97	0.04
NDF	64.5	63.6	0.78	0.26
ADF	63.4	62.2	0.67	0.87

Abbreviations: ADF = acid detergent fiber, BW = body weight, DM = dry matter, NDF = neutral detergent fiber, and SEM = standard error of mean.

**Table 3:** Blood metabolites and rumen fermentation characteristics in beef cattle fed soybean meal and cricket meal as protein sources.

Item	Soybean meal–based diets	Cricket meal–based diet	SEM	*p* value
Blood urea—nitrogen (mg/dL)	12.1	13.5	1.13	0.87
Blood glucose (mg/dL)	75.3	74.6	2.23	0.58
Ruminal pH	6.9	6.9	0.89	0.73
Ruminal temperature (°C)	39.2	39.0	1.56	0.14
Ruminal ammonia—nitrogen (mg/dL)	16.2	18.4	1.14	0.88
Total volatile fatty acid (mmol/L)	97.7	99.1	3.45	0.22
Acetate (%)	67.6	65.3	2.01	0.73
Propionate (%)	24.6	23.8	1.23	0.45
Butyrate (%)	7.7	5.9	0.87	0.74
Rumen microbes (log_10_ cells/mL)				
Bacteria	10.5	10.5	0.95	0.09
Protozoa	6.9	6.9	0.66	0.77
Fugal zoospores	4.4	4.5	0.51	0.98

Abbreviation: SEM = standard error of mean.

**Table 4:** Growth performance of beef cattle fed soybean meal and cricket meal as protein sources.

Item	Soybean meal–based diets	Cricket meal–based diet	SEM	*p* value
Growth performance				
Initial body weight (kg)	148.4	145.6	3.55	0.22
Final body weight (kg)	190.1	210.5	4.78	0.04
Body weight gain (kg)	41.7	64.9	2.66	0.03
Average daily gain (g)	463.3	721.1	20.5	0.02
Feed conversion ratio	9.1	6.1	0.14	0.02

Abbreviation: SEM = standard error of mean.

## Data Availability

The data that support the findings of this study are available from the corresponding author upon reasonable request.
